# North-Western Educator

**Published:** 1847-12

**Authors:** 


### ARTICLE XI.

*North-Western Educator.* A monthly periodical devoted to Education, Literature, and News.

We have received the first and second numbers of this periodical, the publication of which has been recently commenced in Chicago, under the editorial charge of Mr. J. L. Enos.

It is to appear monthly, containing thirty-two pages in each number, at a subscription price of one dollar per annum.

The work makes a good appearance, and its contents are, we should infer from glancing over the table, well made up.

We understand that it is the intention of the editor to devote a portion of its pages to popular physiology. By the way an excellent idea, as it is high time that this branch of science, with comparative and descriptive anatomy, should form a branch of popular education. There is no subject that could be more interesting or instructive than these, to the student of nature, for they bring him at once to the contemplation of her noblest and most perfect work. E.

